# Mental health services use among Canadian Armed Forces members and Veterans: Data from the 2018 Canadian Armed Forces members and Veterans mental health follow-up survey (CAFMVHS)

**DOI:** 10.3389/frhs.2022.954914

**Published:** 2022-08-22

**Authors:** Kate St. Cyr, Aihua Liu, Rachel A. Plouffe, Maede S. Nouri, Callista A. Forchuk, Sonya G. Wanklyn, Brian M. Bird, Deniz Fikretoglu, Alyson L. Mahar, Anthony Nazarov, J. Don Richardson

**Affiliations:** ^1^MacDonald Franklin OSI Research Centre, Lawson Health Research Institute, London, ON, Canada; ^2^Dalla Lana School of Public Health, University of Toronto, Toronto, ON, Canada; ^3^Douglas Mental Health University Institute, Montreal, QC, Canada; ^4^Department of Psychiatry, Western University, London, ON, Canada; ^5^St. Joseph's Operational Stress Injury Clinic, St. Joseph's Healthcare London, London, ON, Canada; ^6^Department of Psychology, Simon Fraser University, Burnaby, BC, Canada; ^7^Defence Research and Development Canada, Toronto Research Centre, Toronto, ON, Canada; ^8^Department of Community Health Sciences, University of Manitoba, Winnipeg, MB, Canada; ^9^Department of Psychiatry and Behavioral Neurosciences, McMaster University, Hamilton, ON, Canada

**Keywords:** mental health services use, military personnel, Veterans, follow up survey, Canadian Armed Forces (CAF)

## Abstract

**Background:**

Differences in healthcare delivery systems and pathways to mental healthcare for Canadian Armed Forces (CAF) members and Veterans may contribute to variations in mental health services use (MHSU) and the factors associated with it. We: (1) estimated the prevalence of past 12-month MHSU (≥1 visit with a medical or mental health professional); and (2) identified sociodemographic, military-, trauma-, and health-related variables associated with MHSU among CAF members and Veterans.

**Methods:**

The current study used data from the 2018 CAF Members and Veterans Mental Health Follow-Up Survey (CAFVMHS). Model variables were selected a priori, and their respective associations with MHSU were estimated among (1) CAF members and (2) Veterans using separate multivariable logistic regression models.

**Results:**

Similar proportions of CAF members and Veterans reported past 12-month MHSU (26.9 vs. 27.5%, respectively). For both CAF members and Veterans, meeting criteria for at least one past 12-month MH disorder was associated with past 12-month MHSU [adjusted odds ratio (AOR) = 7.80, 95% confidence interval (CI) = 7.18–8.46; and AOR = 11.82, 95% CI: 11.07–12.61, respectively). Past-year suicide ideation, a history of sexual trauma, and endorsement of adverse childhood experiences were also significantly associated with MHSU among CAF members and Veterans.

**Significance:**

Similar to previous research, meeting screening criteria for a past 12-month MH disorder was strongly associated with MHSU among both samples. This study extends our existing knowledge about factors associated with MHSU among CAF members and Veterans, and offers direction for future research to increase MHSU.

## Introduction

Military careers carry unique occupational risks, including the possibility of exposure to psychologically distressing events. Previous research has documented elevated rates of mental health (MH) disorders, such as major depressive disorder and posttraumatic stress disorder (PTSD), among military members and Veterans compared to the general population (1–4). A recent review summarizing existing research related to mental health services use (MHSU) across military populations within Australia, Canada, New Zealand, the United Kingdom, and the United States highlighted the role of pathways to care in improving MHSU among military populations (5).

### Pathways to mental healthcare for military populations in Canada

Within Canada, pathways to healthcare, including mental healthcare, differ for Regular Force (RegF) members of the Canadian Armed Forces (CAF) vs. Reserve Force (ResF) members and Veterans of the CAF. While serving in the CAF, RegF members, who are full-time employees of the CAF, receive their healthcare through the Canadian Forces Health Services Group, a CAF-specific, federally-overseen healthcare system (6). Within this system, healthcare—including mental healthcare—is meant to be easily accessible, and regular contact with health services is not uncommon given the physical and mental exertion associated with the nature of a military occupation. For ResF members, who are primarily part-time employees of the CAF, the pathway to healthcare is more complicated. Generally, ResF members receive the majority of their healthcare through the provincially-funded healthcare systems, although they may receive healthcare via the federally-funded CAF healthcare system while deployed or for service-related health conditions (7).

Upon exiting the CAF and transitioning to civilian life, Veterans of the CAF transition to provincial healthcare coverage (8). One of the most notable differences between the federally-funded CAF and civilian provincial healthcare systems is that only select MH services are covered under provincial health insurance. For example, within Ontario - Canada's most populous province - psychotherapy provided by a medical doctor is covered under the provincial health insurance plan, but services provided by other MH professionals, such as psychotherapists, are generally only covered if provided within a provincially-funded hospital or clinic (9). Veterans who go on to other employment following their career with the CAF may have mental healthcare coverage through employee assistance programs, while others may be eligible for Veterans Affairs Canada (VAC) assistance in terms of accessing and funding care for service-related MH conditions (8). Despite this, previous research shows that Veterans may experience difficulties rostering with a primary healthcare provider, who often provide or initiate referrals for mental healthcare, in a timely manner following their release from the CAF (10) or locating a provider who can offer culturally competent care (11, 12). Veterans may also experience challenges navigating the VAC benefits and services that may be available to them (10). These hurdles may exacerbate existing MH symptoms, and could subsequently impact continuity of mental healthcare initiated while still serving in the CAF, or delay the initiation of necessary care.

### MHSU among active CAF members

The first nationally representative MH survey of CAF members, the Canadian Community Health Survey – Canadian Forces Supplement (CCHS-CFS), was conducted in 2002 and revealed a number of important findings, including a higher past 12-month prevalence of depression and panic disorder among CAF RegF members compared to the general Canadian population (13) and a relatively high perceived need for mental healthcare (~30% of respondents) (14), but lower MHSU (11.2% of respondents) in the year preceding survey administration (15). A number of variables associated with past 12-month MHSU, including female sex (15, 16), being older (15), being in the RegF (vs. ResF) (15), having a lower rank (15), and psychiatric need (15), have been identified using CCHS-CFS data. Among respondents who met screening criteria for PTSD on the CCHS-CFS, exposure to multiple types of traumatic experiences (17, 18), type of trauma experienced (17, 18), marital status (18), and income (18) were also associated with past 12-month MHSU.

In the years following the 2002 CCHS-CFS administration, the CAF made significant changes to its MH system, bolstering its capacity to address MH concerns via strategies such as pre- and post-deployment MH screening and improved access to specialized mental healthcare (7). The 2013 Canadian Forces Mental Health Survey (CFMHS) aimed to understand how these systemic changes, combined with the potential impacts of the combat mission in Afghanistan between 2001 and 2014 – Canada's first combat mission since the Korean War – impacted MH and MHSU among CAF ResF members who had deployed in support of the mission in Afghanistan and RegF members. Among personnel who had deployed at least once to Afghanistan, ResF members were less likely to engage in past 12-month MHSU than RegF members, and more likely to report an unmet need for mental healthcare than RegF members (19). Another study found that CAF element (Army, Navy, or Air Force) was not significantly associated with MHSU after accounting for potentially confounding sociodemographic, military-, and health-related variables (20). Importantly, notable increases in the prevalence of MHSU were reported between the CCHS-CFS and CFMHS administrations, potentially indicating that changes made to the CAF's healthcare system during this period of time improved access to mental healthcare (21).

### MHSU among Veterans

Among Veterans, existing research related to MHSU has primarily been conducted using smaller national surveys or population-level data from the province of Ontario. The Life After Service Survey (LASS) is a national survey of Canadian Veterans that was administered in 2013, 2016, and 2019 (22). Findings from these surveys indicate an increase in the number of Veterans reporting (a) MH conditions, such as depression, anxiety and PTSD; and (b) MHSU, between 2016 and 2019 (23). Additionally, a report from the 2019 survey found that both male and female Veterans were more likely to report MHSU than an age-standardized comparison group of male and female civilians (24). Population-level data from Ontario suggests that almost 30% of Veterans had a MH-related encounter with a primary care physician in the first 5 years following their release from the CAF, while almost 6% received care from a psychiatrist and ~2.5% attending an emergency department for MH concerns (25). Findings from this study help elucidate some of the pathways to mental healthcare for Veterans residing in Ontario.

Despite existing research in Canadian military populations, the impact of differential access to mental healthcare between CAF members and Veterans on MHSU remains understudied. The overarching aim of this study was to describe MHSU among Veterans and currently-serving CAF members, using a follow up sample of those who served as CAF RegF members in 2002. The specific objectives of this manuscript are to: (1) assess the proportion of CAF members and Veterans engaging in past 12-month MHSU; and (2) identify sociodemographic, military-, trauma-, and health-related variables associated with MHSU among CAF members and Veterans. Despite a seemingly more accessible pathway to mental healthcare, we hypothesize that CAF members may be more reticent to report MHSU and therefore have a lower reported prevalence of past 12-month MHSU than Veterans; and that for both samples, health-related variables will be most strongly associated with past 12-month MHSU.

## Methods

### Data source

The Canadian Armed Forces Members and Veterans Mental Health Follow-Up Survey (CAFVMHS) is a follow-up survey of current and former members of the CAF who participated in the 2002 CCHS-CFS. Interview data from the in-person, computer-assisted interviews were collected between January 8 and May 31, 2018 (26). Details about the survey methodology have been described comprehensively in the CAFVMHS rationale and methodology paper (27).

### Study population

CAF members and Veterans were eligible to complete the 2018 CAFVMHS if they: (1) were members of the RegF at the time of the 2002 CCHS-CFS; (2) completed the 2002 CCHS-CFS interview; (3) were still alive at the time of data collection in 2018; (4) could be located for the follow-up survey; (5) (resided in one of the ten Canadian provinces; and (6) agreed to have the 2018 CAFVMHS data linked to their 2002 CCHS-CFS data. A small number (*n* = 256) were also randomly selected for exclusion from the CAFVMHS due to budget restrictions; and an additional 1,316 opted out of participating in the CAFVMHS, leaving a total sample size of 2,941 before longitudinal weights provided by Statistics Canada were applied and rounded to a base of 20, resulting in a weighted sample of 18,120 CAF members and 34, 380 Veterans. A previous analysis found the attrition rate among eligible participants between the original 2002 CCHS-CFS survey and the CAFVMHS to be 31.2%, which the authors indicated is somewhat lower than studies in the general population with a similar period of follow up time. Being younger, a member of the Army, and a lifetime history of suicide attempt at baseline were associated with increased odds of attrition, while being female, separated/divorced/widowed, or an officer were associated with exclusion from the follow up survey [reasons for exclusion included random removal due to budgetary constraints, having completed another Statistics Canada-administered survey (e.g., the Life After Service Survey), or having moved outside of the ten Canadian provinces/being unreachable] (28). Approximately two-thirds of the study sample [65.5%; 95% confidence interval (CI) = 63.6–67.3] were Veterans of the CAF in 2018; the remaining 34.5% (95% CI = 32.7–36.4) continued to be actively serving members (28).

### Measures

The current study focuses only on survey items asked in the 2018 CAFVMHS. A number of sociodemographic, military, trauma, and MH-related variables thought to be associated with MHSU were selected a priori for inclusion in the analysis based on findings from previous MHSU-related research among CAF members and Veterans described above.

#### Outcome variable – past 12-month MHSU

Participants were asked whether they had consulted any of the following healthcare professionals for issues related to emotions, MH, or use of alcohol or drugs in the past 12-months: psychiatrist, medical doctor/general practitioner, other medical doctor, psychologist, nurse, and social worker. A derived variable assessing whether respondents sought care related to emotions, MH, or alcohol/drug use from any of these healthcare professionals in the past 12-months was generated; this dichotomous yes/no variable served as the outcome variable for the current analysis.

#### Sociodemographic variables

Age was treated as a dichotomous variable for still-serving CAF members (≤44vs. ≥45 years) and a three-level categorical variable for Veterans (≤44 years, 45–60 years, and 61–75 years). Age categories for CAF members and Veterans varied slightly due to differences in age distributions. We did not include age as a continuous variable in the regression model, primarily due to the large number of parameters to be estimated relative to the expected number of events. Sex was assessed as a dichotomous variable (male vs. female). Marital status was dichotomized as married/common-law vs. widowed/separated/divorced/single. Finally, highest level of education was dichotomized as completed secondary school or less vs. any post-secondary education.

#### Military-related variables

Veteran status was dichotomized based on whether a respondent was currently serving in the CAF as either a RegF or Reserve Force (ResF) member (CAF member) or had released from the CAF (Veteran) at the time of survey administration. Current military component (for actively serving members) and military component at time of release (for Veterans) was dichotomized as RegF vs. ResF. A history of ever being deployed outside of Canada, regardless of mission type, was assessed using a dichotomous yes/no variable for both CAF members and Veterans. Current rank (for CAF members) and rank at time of release (for Veterans) was operationalized as a categorical variable [junior non-commissioned member (NCM), senior NCM, junior officer, and senior officer]; and length of service was assessed as a continuous variable, in years.

#### Trauma-related variables

A history of ever experiencing sexual trauma was assessed with a categorical variable (military-related sexual trauma, non-military-related sexual trauma, and no sexual trauma). Adverse childhood experiences were evaluated using nine questions from the Childhood Experiences of Violence Questionnaire (29). Participants' responses were summed to capture the number of types of adverse events experienced, and then dichotomized as yes/no based on whether they endorsed any adverse childhood experiences. We opted to dichotomize adverse childhood experiences in order to reduce the number of parameters to be assessed in the regression models.

#### MH-related variables

Presence of selected past 12-month MH disorders were assessed using the World Health Organization Composite International Diagnostic Interview (WHO-CIDI), and were derived based on the Diagnostic and Statistical Manual, Fourth Edition (DSM-IV-TR) (30). MH disorders assessed included PTSD, major depressive episodes (MDE), generalized anxiety disorder (GAD), panic disorder (PD), and social phobia (SP). A dichotomous variable capturing whether participants met screening criteria for any of these five past 12-month MH disorders was derived for use in the regression analyses due to concerns about high correlations between MH disorders. Past 12-month suicide ideation (SI) was assessed by asking participants whether they had thought about attempting suicide in the past year, and was dichotomized as yes/no.

### Data analysis

Descriptive statistics (means and standard deviations for continuous variables; frequencies and percentages for categorical variables) were used to describe the sample characteristics by Veteran status. Bivariate analyses in the form of simple logistic regression models and *C* statistics were used to explore the crude associations between each variable of interest and the MHSU outcome.

Multivariable logistic regression models were employed to identify variables independently associated with MHSU among (1) CAF members and (2) Veterans. Model fit was assessed with *C* statistics. All analyses were conducted with longitudinal weights provided by Statistics Canada to provide estimates that were representative of the original 2002 CCHS-CFS sample. 95% CIs were calculated using the 500 bootstrapped weights provided by Statistics Canada.

All statistical analyses were conducted using SAS statistical software, version 9.4 (31).

## Results

### Sample characteristics

Table 1 displays the sample characteristics stratified by Veteran status. Table 2 shows a breakdown of MHSU by Veteran status and type of healthcare provider.

**Table 1:** Sociodemographic, military, and health-related characteristics of 2018 CAFVMHS respondents, by Veteran status.

	**N (%; 95% CI)**	
**Variable**	**Current CAF members** **(weighted *n* = 18,120)**	**Veterans** **(weighted *n* = 34,380)**
Sex		
Male Female	15,860 (87.5; 86.3– 88.8) 2,260 (12.5;11.2–−13.7)	30,260 (88.0; 87.4–−88.6) 4,120 (12.0;11.4–−12.6)
**Age**		
≤ 44 years 45 to 60 years 61 to 75years	6,640 (36.6; 33.1–40.2) 11,480 (63.4; 59.8–66.9)* -	4,400 (12.8; 11.0–14.6) 24,380 (70.9; 68.7–73.2) 5,600 (16.3;14.9–17.7)
**Marital status**		
Married Common-law Widowed/divorced/separated Single (nevermarried)	12,080 (67.0;63.7–70.3) 3,200 (17.7;14.9–20.5) 1,720 (9.5;7.4–11.7) 1,040 (5.76;4.04–7.49)	23,900 (69.8; 67.6–72.0) 4,080 (11.9; 10.3–13.5) 4,020 (11.7; 10.1–13.4) 2,240 (6.5; 5.3–7.8)
**Education**		
Completed secondary or less Post-secondary orhigher	7,900 (43.8; 40.2–47.4) 10,140 (56.2;52.6–59.8)	14,480 (42.3; 40.0–44.7) 19,740 (57.7;55.3–60.1)
**Military component**		
RegF ResF	15,440 (85.2; 8.30–87.5) 2,680 (14.8;12.5–17.1)	30,520 (88.9; 87.5–90.2) 3,820 (11.1;9.8–12.5)
**Rank**		
Junior NCM Senior NCM Junior officer Seniorofficer	2,220 (12.3; 9.7–14.8) 9,800 (54.1; 50.7–57.5) 1,600 (8.8; 7.00–10.7) 4,500 (24.8;22.4–27.2)	11,680 (34.0; 31.7–36.2) 15,320 (44.6; 42.4–46.8) 2,800 (8.1; 7.0–9.3) 4,580(13.3;12.3–14.3)
**Years of service**		
1–21 22–27 28–33 34–47	5,420 (29.9; 26.5–33.4) 4,200 (23.2; 20.2–26.2) 5,540 (30.6; 27.4–33.9) 2,920 (16.1;13.9–18.3)	9,840 (28.7; 26.3 – 31.0) 10,640 (31.0; 28.6–33.4) 7,180 (20.9; 19.1–22.7) 6,680 (19.5; 18.0 –21.0)
**Deployed outside of Canada**		
Yes No	16,080 (88.7; 86.6–90.9) 2,020 (11.2;9.0–13.3)	27,600 (80.3; 78.5–82.1) 6,780 (19.7;17.9–21.5)
**Any past 12-month MH disorder**		
Yes No	4,600 (25.6; 22.3–28.9) 13,380 (74.4;71.1–77.7)	11,000 (32.7; 30.4–35.0) 22,640 (67.3;65.0–69.7)

^*^Collapsed into a single category (e.g., 45+ years) due to small cell sizes in the 60–75 year category among CAF members.

**Table 2:** Frequency and percentage of past 12-month mental health services use, by Veteran status and healthcare provider type.

	***N*** **(%; 95% CI)**	
	**Current CAF members ** **(weighted *n* = 18,120)**	**Veterans** **(weighted *n* = 34,380)**
Psychiatrist	1,440 (8.0; 5.9–10.0)	3,180 (9.3; 7.8–10.8)
Family doctor	2,920 (16.1; 13.5–18.7)	6,360 (18.5; 16.6–20.5)
Other medical doctor	500 (2.8; 1.6–3.9)	1,020 (3.0; 2.1–3.8)
Psychologist	2,300 (12.7; 10.3–15.1)	5,500 (16.0; 14.1–18.0)
Nurse/physician assistant	1,580 (8.7; 6.7–10.8)	1,460 (4.3; 3.2–5.3)
Social worker	2,960 (16.3; 13.6–19.1)	2,440 (7.1; 5.8–8.4)

### Prevalence of MHSU by Veteran status

Similar proportions of CAF members and Veterans endorsed having at least one MH-related visit with a healthcare professional in the past 12 months (CAF members: weighted *n* = 4,880; 26.9%, 95% CI = 23.7–30.2; Veterans: weighted *n* = 9,460; 27.6%, 95% CI = 25.4–29.8). A slightly higher proportion of Veterans reported engagement in past 12-month MHSU with a psychiatrist or psychologist, whereas almost twice as many active CAF members endorsed MHSU with a nurse/physician assistant or social worker compared to Veterans (see Table 2 for frequencies and percentages).

### Bivariate analyses

Among current CAF members, all identified sociodemographic, military, trauma, and MH-related variables were significantly associated with MHSU except for deployment out of Canada [crude odds ratio (OR) = 1.01, 95% CI = 0.91–1.13, *p* = 0.80] (see Table 3). Among Veterans, all variables were significantly associated with MHSU.

**Table 3:** Crude odds ratios of the associations between sociodemographic, military-, trauma-, and health-related variables and past 12-month mental health services use, by Veteran status.

	**Current CAF members**		**Veterans**	
	**OR (95% CI)**	***p*-value**	**OR (95% CI)**	***p*-value**
**Demographics**		
Age		
< =44 vs. 45+ < =44 vs. 61–75 45–60 vs. 61–75	1.22 (1.14–1.31)* - -	<0.0001 - -	- 5.72 (5.16–6.33) 3.16 (2.90–3.45)	- <0.0001 <0.0001
Sex (ref = male)		
Female	1.25 (1.14–1.38)	<0.0001	1.59 (1.49–1.71)	<0.0001
Highest education level (ref = secondary or less)		
More than secondary	0.79 (0.74–0.84)	<0.0001	1.11 (1.06–1.16)	<0.0001
Marital status (ref = widowed/separated/divorced/single)		
Married/common-law	1.57 (1.44–1.71)	<0.0001	1.63 (1.53–1.72)	<0.0001
**Military factors**		
Military component (ref = ResF)		
RegF	0.43 (0.38–0.48)	<0.0001	0.41 (0.38–0.45)	<0.0001
Ever deployed out of Canada (ref = no)		
Yes	1.01 (0.91–1.13)	0.8039	2.35 (2.19–2.52)	<0.0001
Length of service (years)	0.96 (0.96–0.97)	<0.0001	0.96 (0.96–0.96)	<0.0001
Rank (ref: Senior officer)		
Junior NCM Senior NCM Junior officer	2.16 (1.94–2.41) 1.49 (1.36–1.63) 1.24 (1.08–1.43)	<0.0001 <0.0001 0.0022	2.47 (2.27–2.70) 1.67 (1.53–1.82) 1.71 (1.52–1.92)	<0.0001 <0.0001 <0.0001
Sexual assault or unwanted sexual touching (ref: no trauma)		
Military-related Non-military-related	1.83 (1.66–2.02) 3.54 (3.03–4.12)	<0.0001 <0.0001	2.72 (2.52–2.94) 2.33 (2.17–2.51)	<0.0001 <0.0001
**Past 12-month mental health status**		
Past 12-month MDE (ref = no) Yes Past 12-month PD (ref = no) Yes Past 12-month GAD (ref = no) Yes Past 12-month PTSD (ref = no) Yes Past 12-month SP (ref = no) Yes Any past 12-month MH disorder (ref = no) Yes Past 12-month suicide ideation (ref = no) Yes	14.05 (12.81−15.41) 11.15 (9.84–12.63) 13.20 (11.44–15.22) 4.15 (3.77–4.56) 9.88 (8.87–11.01) 9.47 (8.77–10.22) 7.78 (6.71–9.01)	<0.0001 <0.0001 <0.0001 <0.0001 <0.0001 <0.0001 <0.0001	13.21 (12.44–14.04) 10.97 (10.18–11.82) 30.13 (27.25–33.33) 13.2 (12.34–14.11) 10.39 (9.71–11.11) 17.15 (16.17–18.19) 7.57 (7.02–8.17)	<0.0001 <0.0001 <0.0001 <0.0001 <0.0001 <0.0001 <0.0001
**Adverse childhood experiences**		
Any adverse childhood experiences (ref = no) Yes	1.78 (1.66 – 1.91)	<0.0001	1.90 (1.80 – 2.00)	<0.0001

^*^Collapsed into a single category (e.g., 45+ years) due to small cell sizes in the 60-75 year category among CAF members.

### Multivariable logistic regression models assessing associations with MHSU

The multivariable logistic regression models for both CAF members and Veterans included age, sex, marital status, highest level of education, RegF vs. ResF, deployed out of Canada status, sexual trauma (military-related and non-military-related), any adverse childhood experience, past 12-month SI, and any past 12-month MH disorder. Rank and length of military service were removed due to multicollinearity issues with age. The *C*-statistic for the final CAF member model was 0.771, whereas the *C*-statistic for the final Veteran model was 0.847. Models are typically considered a reasonable fit when the *C*-statistic is higher than 0.7 and a good fit when the *C*-statistic is higher than 0.8 (32). The results of the logistic regression models are displayed in Table 4. Among Veterans, all variables remained statistically significant in the multivariable regression model, while highest level of education, deployment outside of Canada, and age were no longer statistically significant after inclusion in the multivariable regression model for CAF members. Among both CAF members and Veterans, meeting criteria for any past 12-month MH condition (MDE, PD, SP, GAD, or PTSD) had a stronger association with past 12-month MHSU than any other sociodemographic, military-, trauma-, or health-related variable (CAF members: AOR = 7.80, 95% CI = 7.18–8.46; Veterans: AOR = 11.82, 95% CI = 11.07–12.61). Past 12-month SI was also strongly and significantly associated with past 12-month MHSU (CAF members: AOR = 2.37, 95% CI = 2.00–2.80; Veterans: AOR = 2.18, 95% CI = 1.98–2.39). CAF members with a history of military-related and non-military-related sexual trauma had 1.49 (95% CI = 1.30–1.71) and 1.61 (95% CI = 1.41–1.83) times the odds, respectively, of reporting past 12-month MHSU compared to CAF members with no history of sexual trauma; Veterans with a history of military-related and non-military related had 1.27 (95% CI = 1.14–1.43 and 1.15–1.40, respectively) times the odds of reporting past 12-month MHSU compared to Veterans without a history of sexual trauma. Finally, history of adverse childhood experiences was also significantly associated with increased odds of past 12-month MHSU among CAF members (AOR = 1.42, 95% CI = 1.30–1.54) and Veterans (AOR = 1.33, 95% CI = 1.25–1.42).

**Table 4:** Adjusted odds ratios of the association between past 12-month mental health services use, by Veteran status.

	**Current CAF members**		**Veterans**	
	**AOR (95% CI)**	***p***	**AOR (95% CI)**	***p***
**Age**				
< =44 vs. 45+ < =44 vs. 61–75 45–60 vs. 61–75	0.97 (0.89–1.05)* - -	0.3993 - -	- 3.26 (2.87–3.70) 2.26 (2.04–2.51)	- <0.0001 <0.0001
**Sex (ref** **=** **male)**				
Female	1.03 (0.90–1.18)	0.6899	1.54 (1.39–1.58)	<0.0001
**Education (ref** **=** **secondary or less)**				
More than secondary	0.94 (0.87–1.02)	0.1479	1.48 (1.39–1.71)	<0.0001
**Marital status (ref** **=** **widowed/separated/divorced/single)**				
Married/common-law	1.14 (1.02–1.26)	0.0158	1.49 (1.38–1.61)	<0.0001
**Military component (ref** **=** **ResF)**				
RegF	0.50 (0.44–0.57)	<0.0001	0.69 (0.62–0.77)	<0.0001
**Ever deployed out of Canada (ref** **=** **no)**				
Yes	0.95 (0.83–1.07)	0.3855	2.08 (1.90–2.28)	<0.0001
**Any sexual assault/unwanted touching (ref** **=** **no)**				
Military-related Non-military-related	1.49 (1.30–1.71) 1.61 (1.41–1.83)	<0.0001 <0.0001	1.27 (1.14–1.43) 1.27 (1.15–1.40)	<0.0001 <0.0001
**Any adverse childhood experiences (ref** **=** **no)**				
Yes	1.42 (1.30–1.54)	<0.0001	1.33 (1.25–1.42)	<0.0001
**Any past 12-month mental health disorder (ref** **=** **no)**				
*Yes*	7.80 (7.18–8.46)	<0.0001	11.82 (11.07–12.61)	<0.0001
**Any past 12-month suicide ideation (ref** **=** **no)**				
Yes	2.37 (2.00–2.80)	<0.0001	2.18 (1.98–2.39)	<0.0001

^*^Collapsed into a single category (e.g., 45+ years) due to small cell sizes in the 60–75 year category among CAF members.

## Discussion

Despite differences in pathways to mental healthcare, similar proportions of active CAF members and Veterans – slightly more than one-quarter – reported engaging in past 12-month MHSU. A higher proportion of CAF members reported past 12-month MHSU with a nurse/physician assistant or social worker than Veterans, which is likely a reflection of care providers and pathways within the CAF healthcare system. Among both the CAF member and Veteran models, meeting criteria for any past 12-month MH disorder was strongly associated with MHSU. This finding is similar to previous research exploring predictors of MHSU among active CAF members (15). Past-year SI, a history of sexual trauma, and endorsement of any adverse childhood experiences were also significantly associated with MHSU among CAF members and Veterans, though to a lesser extent than past 12-month MH disorders. Given the potentially traumatic nature of these experiences, and their association with poor MH outcomes in various populations (33, 34), it is reasonable to assume a positive association between their occurrence and the likelihood of engaging in MHSU.

Deployment out of Canada was significantly associated with MHSU among Veterans but not CAF members in both the crude and adjusted regression models. This finding may be at least partially attributable to the healthy soldier effect (35). Specifically, CAF members who experience negative psychological sequelae following deployment on a humanitarian, peacekeeping, or combat mission might be more likely to release from the CAF and subsequently seek MHSU, whereas individuals who deployed overseas and remained in CAF may not differ in terms of need for MHSU compared to those who did not deploy outside of Canada. Alternatively, this finding may be the result of facilitated access to mental healthcare within the CAF healthcare system.

After including all variables in the regression model, age, sex, and highest level of education were statistically significantly associated with MHSU among Veterans, but not CAF members. The non-significant finding for sex among CAF members in the multivariable model was likely due to collinearity between sex, age, education, and other variables included in the model, as these variables were all significant in the simple logistic regression models. In the absence of significant collinearity, a theoretical explanation could be that sex-specific differences in health-seeking behaviors are minimized in still-serving populations, where contact with health services is common (36); and that once released, the sociocultural tendencies of women to engage more frequently in healthcare-seeking behaviors than their male counterparts may become more apparent (37). The non-significant findings for age among CAF members may be partially a result of the restricted age range (e.g., fewer older adults than in the Veteran population), as well as the healthy soldier effect; while the non-significant findings for education may, like sex, be associated with healthcare-seeking behaviors.

In the adjusted model, RegF vs. ResF membership was not significantly associated with MHSU among either the active CAF or Veteran subsample. This is somewhat different than previous research, which has demonstrated lower likelihood of MHSU among ResF members compared to RegF members (19). Within the current study, the non-significant finding may be a function of all study participants being RegF members in 2002; as such, their occupational experiences, particularly among ResF members who were deployed to the combat mission in Afghanistan, may not differ significantly from RegF members.

### Strengths and limitations

Our study had a number of notable strengths. First, it included a reasonably large follow-up sample of CAF members and Veterans. The use of survey weights also helped to ensure the generalizability of our findings to the original CAF RegF military population in 2002. The survey design also allowed us to investigate MHSU within current CAF members and Veterans who served during a similar period of time (i.e., all were RegF members of the CAF in 2002). Additionally, the computer-assisted structured interview used in this study was designed to aid in recall and ensure interview questions were asked in a standardized manner, which reduced the likelihood of interviewer bias.

In terms of limitations, it is possible that individuals who declined participation in the CAFVMHS differed from those who did agree to participate, although a previous study exploring attrition between the CCHS-CFS and CAFMVHS surveys found no associations between military status, MH conditions, traumatic experiences, adverse childhood experiences, and attrition (28). This particular study did not explore participants' perceived need for mental healthcare, nor were participants asked about factors that may have facilitated or prevented access to mental healthcare, which could help explain our findings and identify opportunities to increase MHSU. We recognize that there are likely other variables that may be associated with MHSU, such as rank, CAF element (e.g., Army, Navy, and Air Force), and income that were not included in the analyses here due to limitations associated with the number of “events” [i.e., number of respondents who reported past 12-month MHSU) within each subsample and the number of parameters to be estimated (e.g., the “events per variable” (EPV) rule] (38, 39). We dichotomized several variables (e.g., adverse childhood experiences) because of similar concerns about the EPV rule, potentially resulting in a loss of information. It is also possible that the factors associated with MHSU might vary by type of mental healthcare provider. However, we were unable to explore each provider type individually due to small cell sizes. A qualitative component asking military members and Veterans about the types of barriers and facilitators to receiving culturally competent mental healthcare may have aided in the interpretation of our findings. Finally, because of sample size limitations, we were unable to explore factors associated with MHSU among currently serving and former ResF members. Including these individuals would reveal important knowledge that could help inform mental healthcare access strategies for ResF members, and should be sought out in future research.

## Conclusions

The current study explored MHSU across CAF members and Veterans, and assessed variables associated with MHSU among these subsamples. We found that similar proportions of CAF members and Veterans reported engaging in past 12-month MHSU; and that meeting screening criteria for a past 12-month MH disorder was more strongly associated with MHSU among both samples than any of the other sociodemographic, military-, trauma-, and health-related variables considered in the analysis. Demographic variables, such as sex and highest level of education attained, were associated with MHSU only among the Veteran subsample. This finding may help identify potential avenues for future research related to understanding facilitators and barriers to mental healthcare when accessing it outside of the internal CAF healthcare system, such as access and attitudes toward mental healthcare. This study also extends our existing knowledge about variables associated with MHSU, which offers direction for future research to identify opportunities to increase MHSU. Future research may also benefit from oversampling ResF members so that variables associated with MHSU can be assessed within this subpopulation, and from asking CAF members and Veterans specifically about their health-seeking behaviors and the factors that increase or reduce their access to mental healthcare.

## Data availability statement

The data analyzed in this study are subject to the following licenses/restrictions: Data are not publicly available, but access may be obtained from a Statistics Canada Research Data Centre. Requests to access the datasets should be directed to Statistics Canada's Statistical Information Service at STATCAN.infostats-infostats.STATCAN@canada.ca.

## Ethics statement

The studies involving human participants were reviewed and approved by Statistics Canada. The patients/participants provided their written informed consent to participate in this study.

## Author contributions

KS and AN conceptualized the study, while KS, AN, AL, RP, MN, SW, and BB conceptualized the analysis plan. AL had access to the data and conducted the analysis. KS drafted the initial manuscript, which was critically reviewed by all authors. All authors reviewed the analytic output and contributed to the interpretations and read and approved the final manuscript as submitted and agree to be accountable for all aspects of the work.

## Conflict of interest

The authors declare that the research was conducted in the absence of any commercial or financial relationships that could be construed as a potential conflict of interest.

## Publisher's note

All claims expressed in this article are solely those of the authors and do not necessarily represent those of their affiliated organizations, or those of the publisher, the editors and the reviewers. Any product that may be evaluated in this article, or claim that may be made by its manufacturer, is not guaranteed or endorsed by the publisher.
